# Comparison of the Supraclavicular, Infraclavicular and Axillary Approaches for Ultrasound-Guided Brachial Plexus Block for Surgical Anesthesia

**DOI:** 10.5041/RMMJ.10240

**Published:** 2016-04-19

**Authors:** Anatoli Stav, Leonid Reytman, Michael-Yohay Stav, Isaak Portnoy, Alexander Kantarovsky, Offer Galili, Shmuel Luboshitz, Roger Sevi, Ahud Sternberg

**Affiliations:** 1 Postanesthesia Care Unit, Hillel Yaffe Medical Center, Hadera, Israel; 2 The Ruth and Bruce Rappaport Faculty of Medicine, Technion–Israel Institute of Technology, Haifa, Israel; 3 Department of Anesthesiology, Hillel Yaffe Medical Center, Hadera, Israel; 4 Vascular Surgery Unit, Hillel Yaffe Medical Center, Hadera, Israel; 5 Orthopedic Hand Surgery Unit, Hillel Yaffe Medical Center, Hadera, Israel; 6 Department of Orthopedics A, Hillel Yaffe Medical Center, Hadera, Israel; 7 Department of Surgery A, Hillel Yaffe Medical Center, Hadera, Israel

**Keywords:** Axillary, brachial plexus block, infraclavicular, regional anesthesia, supraclavicular, surgical anesthesia, ultrasound-guided

## Abstract

**Objective:**

We hypothesized that ultrasound (US)-guided technique of the supra- and infraclavicular and axillary approaches of brachial plexus block (BPB) will produce a high quality of surgical anesthesia for operations below the shoulder independently of the approach and body mass index (BMI). Intercostobrachial and medial brachial cutaneous nerves will be blocked separately because they are not a part of the brachial plexus.

**Methods:**

This is a prospective randomized observer-blinded study. The three approaches of the US-guided BPB without neurostimulation were compared for quality, performance time, and correlation between performance time and BMI. Intercostobrachial and medial brachial cutaneous nerve blocks were used in all patients.

**Results:**

A total of 101 patients were randomized into three groups: SCL (supraclavicular), ICL (infraclavicular), and AX (axillary). Seven patients were excluded due to various factors. All three groups were similar in demographic data, M:F proportion, preoperative diagnosis and type of surgery, anesthesiologists who performed the block, and surgical staff that performed the surgical intervention. The time between the end of the block performance and the start of the operation was also similar. The quality of the surgical anesthesia and discomfort during the operation were identical following comparison between groups. No direct positive correlation was observed between BMI and the block performance time. The time for the axillary block was slightly longer than the time for the supra- and infraclavicular approaches, but it had no practical clinical significance. Transient Horner syndrome was observed in three patients in the SCL group. No other adverse effects or complications were observed.

**Conclusions:**

All three approaches can be used for US-guided BPB with similar quality of surgical anesthesia for operations of below the shoulder. A block of the intercostobrachial and medial brachial cutaneous nerves is recommended. Obesity is not a significant factor in relation to the time of US-guided BPB performance, or the quality of surgical anesthesia. (ClinicalTrials.gov number, NCT01442558.)

## INTRODUCTION

When a nerve block is used as a surgical anesthetic, the criteria for assessing the quality of the nerve block are the need of supplementation with another analgesic or the need of conversion to general anesthesia.^1^ The supraclavicular approach to brachial plexus block (SCL) is indicated for operations of the upper extremity distal to the shoulder^2^; the infraclavicular block (ICL) is indicated for operations of the distal arm, elbow, wrist, and hand^3^; and the axillary block (AX) is indicated for surgery of the elbow, forearm, and hand.^4^ There are three methods of performing a regional block: the landmark-guided method, with or without neurostimulation, and the ultrasound-guided technique. The ultrasound (US)-guided technique gives the best quality of regional block, irrespective of the approach, most probably due to the visualization of the target structures (nerve or sheath, or interfascial space, for example), as well as the visualization of the needle and the spread of the local anesthetic after the injection.^5^–^16^ This prospective randomized observer-blind study compares the supraclavicular, infraclavicular, and axillary approaches for US-guided brachial plexus block without nerve stimulation.

## METHODS

After Institutional Review Board approval and written informed consent, 101 patients, older than 18, with American Society of Anesthesiologists (ASA) score I–III, undergoing elective orthopedic or vascular surgery at the level below the axilla and distally, were prospectively enrolled. Patients who suffered from ulnar nerve entrapment were evaluated by electromyography preoperatively. Body mass index (BMI) was recorded for all patients. Exclusion criteria: patients younger than 18 years old; severe chronic obstructive or restrictive lung diseases with continuous treatment with continuous positive airway pressure (CPAP) and/or oxygen; pregnancy; skin infection located near the block injection site; allergy to local anesthetics; preoperative continuous peripheral sensory or motor deficit of the upper limb to be operated on; patients with coagulopathy, international normalized ratio (INR)>1.4, thrombocytopenia (platelet count <100,000), proven opioid dependency, chronic pain syndrome, dementia, and lack of orientation to person, place, and time. Patients with mild sensory deficit due to ulnar nerve entrapment were not excluded from the study. Patients who declined the possibility of having an operation under regional anesthesia and insisted on general anesthesia only, and those in whom a language barrier precluded informed consent were also excluded.

Randomization was done using a randomization program on the internet (randomization.com) on the morning before surgery. Each patient was included in one of the three evaluated groups: SCL, ICL, and AX. If an ipsilateral subclavian vein port or peripherally inserted central catheter (PICC line) had been previously inserted for temporary hemodialysis treatment and it was impossible to perform ICL block, randomization was performed with two possibilities only: SCL and AX.

Patients were premedicated with 0.5–1 μg/kg fentanyl and 2–3 mg IV midazolam. Standard ASA monitoring and supplemental oxygen (mask 40%, 5 liters per minute) were applied throughout the block.

All US-guided blocks were performed, without additional neurostimulation, by one of two experienced anesthesiologists (A. Stav or L.R.). The S-NERVE ultrasound machine (SonoSite Inc., Bothel, WA, USA) with Linear Probe HFL 38x/6–13 MHz was used for visualization of the anatomical structures in all patients. A SonoTAP cannula (PAJUNK GmbH Medizintechnologie, Geisingen, Germany) 22-G 50 mm (in patients of SCL and AX groups) or 80 mm (in patients of ICL group) was used in all patients.

All blocks were performed with 40 mL of bupivacaine 0.5% with adrenaline 1:200,000. Operations were started at least 30 min after the injection of local anesthetic. The SCL block was carried out according to the Jack Vander Beek technique,^17^ ICL according to the Sandhu and Capan technique,^18^ and AX according to Jack Vander Beek technique.^19^

Additional nerves were blocked by subcutaneous local infiltration with lidocaine 1% in the axilla. A hemi-ring injection of lidocaine 1% was used in all patients to eliminate tourniquet pain and pain in the area of distribution of intercostobrachial (Th2) and medial brachial cutaneous (Th1 and Th2) nerves.^20^

The duration of each block procedure was measured from the time of sterile skin preparation by alcohol application to the termination of the injection of local anesthetic and removal of the block needle. The time that was needed for additional block of the intercostobrachial and medial brachial cutaneous nerves was not included.

Sensory and motor block was assessed 30 minutes after the end of the procedure. The assessor was blinded to the approach used for the brachial plexus block. Before the operation was started, loss of sensation was evaluated again by pin-prick with surgical pincers. The surgeon was also blinded to the approach used.

The block was considered as “appropriate” or “failed.” Definition of a failed block was as follows: necessity of a significant addition of strong opioid analgesic, added to the general anesthesia, or to the local infiltrative anesthesia.

The percentage of failed blocks in each group was calculated and compared among groups. This is the primary end-point of the study.

The patients were evaluated 24–36 hours postoperatively until restoration of sensory and motor function of the limb. The variables that were statistically analyzed included the following:

1. Demographic values: age, gender, height (h), body weight (BW). Calculation of body mass index (BMI) was carried out according to formula BMI = BW (kg)/h^2^ (m^2^). The correlation between BMI and block performance time was calculated and compared statistically among the groups.
2. Diagnosis and type of operation. Proportion of orthopedic:vascular operations (arterio-venous fistula creation).
3. Block performance time.
4. The time between the end of the block performance and start of the operation.
5. Duration of the operation.
6. Discomfort during the operation that was not pain (for example discomfort due to prolonged position on the operating table).
7. “Appropriate” or “failed” block.
8. Complications (pneumothorax, temporary ipsilateral hemidiaphragmatic paresis with dyspnea and elevated hemidiaphragm, accidental vascular puncture, local anesthetic toxicity), as well as side effects (Horner syndrome, transient (for more than three days) postoperative neurologic deficit).

## STATISTICAL ANALYSIS

Numerical parameters were analyzed by the Shapiro–Wilk test for normality of distribution. The one-way ANOVA *t* test was used if distribution was normal, and the Kruskal–Wallis test was used in cases of abnormal distribution of the variable, for comparison among categorical variables. The Mann–Whitney *U* test was used for *post hoc* analysis in multiple comparisons. The Fisher exact test, two-tailed *P* value calculation, computed only for 2×2 table, was used for comparison of proportion of categorical variables among the groups. *P*<0.05 was considered as the statistically significant level.

Pearson correlation was used for assessing correlation between BMI and block performance time in each group.

## RESULTS

A total of 101 patients were included in the trial. Seven of them were excluded for various reasons: e.g. start of general anesthesia before evaluation of the quality of peripheral nerve block (error of the anesthesiologist); the block was performed, but the operation was postponed because of an emergency case; back pain and inability of the patient to remain in supine position during the operation.

Variables of 94 patients were included and analyzed statistically: 37 in the SCL group, 23 in the ICL group, and 34 in the AX group. There was no statistically significant difference among the groups in the demographic data, proportion M:F, the time between the end of the block performance and the start of the operation, the duration of the operation (Table 1), the proportion of orthopedic:vascular operations (Table 2). The same type of orthopedic and vascular surgery was performed in all three groups (Table 3). There was no statistically significant difference in the proportion of arterio-venous fistula creation with and without graft among groups (Table 4).

**Table 1:** Demographic Variables.

	SCL (*n*=37)	ICL (*n*=23)	AX (*n*=34)	*P* value
Age (years) *	63.62±14.77	63.00±21.55	60.71±16.42	0.45
Height (meter) †	1.65±0.09	1.66±0.09	1.68±0.09	0.22
Body weight (kg) *	81.51±19.99	73.65±15.46	85.12±21.49	0.063
BMI (kg/m^2^) *	30.22±7.63	23.47±4.35	29.96±6.06	0.081
Gender (M : F) ‡	15 : 22	13 : 10		0.29
Gender (M : F) ‡		13 : 10	20 : 14	1.00
Gender (M : F) ‡	15 : 22		20 : 14	0.16
ASA physical status (I/II/III)	7/1/29	4/4/15	6/3/25	
Time between end of block performance and start of the operation (min) *	91.62±52.07	75.52±60.58	78.32±47.91	0.23
Duration of the operation (min)	73.62±32.45	68.09±34.08	74.85±33.98	0.82

The values are mean±SD.

* Abnormal distribution minimally in one group; Kruskal–Wallis test was used as a mathematical extension of Mann–Whitney–Wilcoxon test.

† Normal distribution, Levene’s test of homogeneity; one-way ANOVA test between groups was used.

‡ Fisher exact test. Two-tailed *P* value calculation, computed only for 2×2 table. Comparison of proportion between two groups.

AX, US-guided brachial plexus block performed by axillary approach; ICL group, US-guided brachial plexus block performed by infraclavicular approach; SCL group, US-guided brachial plexus block performed by supraclavicular approach.

**Table 2:** Comparison of Proportion Orthopedic Operation : Vascular Operation Between Groups.

	SCL (*n*=37)	ICL (*n*=23)	AX (*n*=34)	*P* value
Ort : Vasc	11 : 26	10 : 13		0.40
Ort : Vasc	11 : 26		9 : 25	0.80
Ort : Vasc		10 : 13	9 : 25	0.25

Fisher exact test. Two-tailed P value calculation, computed only for 2×2 table.

Ort : Vasc, proportion of orthopedic : vascular operations. Brachial-axillary access with graft was included too.

**Table 3:** Diagnosis and Type of Orthopedic Surgery.

	Diagnosis	Type of Surgery
SCL	Navicular bone fracture	Resection of styloid process
	Tear of tendon	Suturing of the tendon
	Bursitis of olecranon	Bursectomy
	Recurrent carpal tunnel syndrome	Carpal tunnel release
	Fracture of olecranon (×2)	ORIF
	Osteoarthritis	Carpo-metacarpal arthrodesis
	Scaphoid–radius joint osteoarthritis	Wrist fusion
	Tendinitis	Tendon release
	Fracture of finger with tear of ligaments	ORIF and ligamentorrhaphy

ICL	Tennis elbow (×3)	Hohmann operation; epicondylectomy (×2)
	Fracture of radius and ulna (×2)	ORIF
	Contracture of palmar fascia	Release of the fascia
	Ganglion of the hand	Excision
	Ulnar nerve entrapment, CTS *	Ulnar nerve transposition, CT release
	Trauma; partial tear of the ligament was diagnosed in the elbow region	Ligament reconstruction
	Rheumatoid arthritis	Carpo-carpal and carpo-metacarpal arthrodesis

AX	Ganglion of the hand	Excision
	Olecranon bursitis	Bursectomy
	Ulnar nerve compression *	Ulnar nerve transposition
	Tumor of hand	Excision
	Tear of ligament	Suturing
	Fracture of ulna	ORIF
	Ulnar nerve entrapment *	Ulnar nerve transposition
	Tennis elbow	Osteotomy
	Fracture of metacarpal	ORIF

* Electromyography was performed before the operation.

CT, carpal tunnel; CTS, carpal tunnel syndrome; ORIF, open reduction, internal fixation.

**Table 4:** Proportion of Aterio-Venous Fistula Creation With Graft Gortex : Without Graft.

	SCL	ICL	AX	*P*
With Graft Gortex : Without Graft	12 : 14	4 : 9		0.49
With Graft Gortex : Without Graft	12 : 14		11 : 14	1.00
With Graft Gortex : Without Graft		4 : 9	11 : 14	0.50

Fisher exact test. Two-tailed P value calculation, computed only for 2×2 table.

The duration of the axillary block performance (25.35±9.65 min) was significantly longer than the other two approaches. There was no difference between the SCL (18.32±6.27 min) and ICL (19.48± 7.88 min) groups.

Seven patients in SCL group, seven in ICL group, and 12 in AX group felt some discomfort due to mild pain or other reasons (feeling of cold due to low temperature in the operating room, anxiety before surgery, inconvenient position on the operating table, etc.). An injection of 1–2 mg of midazolam IV or 20–30 mg of propofol usually helped to alleviate these unpleasant sensations. There was no statistically significant difference between groups concerning feelings of discomfort. Three patients from the SCL group, three from the ICL group, and four from the AX group received 3–5 mL of lidocaine 10 mg/mL intra- and subcutaneously due to a positive pin-prick test performed by the surgeon. The patients experienced no pain following skin incision up to the end of surgery. However, those 10 blocks were classified as “failed.” Two patients from the AX group received fentanyl 50–150 μg with midazolam 3–5 mg IV by titration due to moderate pain during surgery. Both of these blocks were also classified as “failed.” One patient from the AX group felt pain in the area innervated by the ulnar nerve; general anesthesia was therefore used. There was no difference between groups in the proportion of “appropriate” to “failed.”

No direct positive correlation was observed between BMI and block performance time (*r*<0.7) in any of the three groups.

Horner syndrome (ptosis, myosis, enophthalmus) was observed in three patients in the SCL group, resolving within 24 hours in all three cases. There were no other adverse effects or complications in any group, and no neurologic deficit was diagnosed in any patient 24–36 hours postoperatively.

## DISCUSSION

General anesthesia is a more popular method of surgical anesthesia in comparison with regional anesthesia (RA), especially in small hospitals.^1^ But RA and especially peripheral nerve blocks provide superior pain control in the immediate postoperative period.^1^ In the era of ultrasound-guidance the peripheral nerve block is a safe,^21^ highly effective,^1^,^22^ minimally invasive,^1^ and cost-effective^23^ method of anesthesia.

All three approaches for brachial plexus block (BPB) evaluated in this study are safe and can be proposed to the patient before an emergency or elective surgery below the level of the shoulder. From previous publications it is known that the SCL block is used for any surgery of the arm distal to the shoulder,^2^,^24^ while ICL block is used for operations distal to the axilla,^3^,^24^ and AX block is used for any surgery on the elbow or distally.^4^,^24^ An additional block of the intercostobrachial and medial brachial cutaneous nerves by intradermal or subcutaneous infiltrations in the axilla or hemi-ring immediately distal to the shoulder^20^ enables performance of any surgery of the arm distal to the shoulder after the infraclavicular or axillary approaches to brachial plexus block. Because the three approaches to BPB that were compared in this study did not differ in the quality of surgical anesthesia, we use any one of the three evaluated approaches for any surgery below the shoulder (distally from site of injection of local anesthetic around target nerves). In all patients we add a block of the intercostobrachial and medial brachial cutaneous nerves.

Block performance time was similar in the SCL and the ICL groups, but block performance time in the AX group was longer. This difference of 5–10 minutes has but small clinical importance. In addition, no significant correlation was found between the time needed for block performance in any of the approaches and BMI. Therefore, US-guided BPB can be performed in obese patients with similar speed as in patients with normal BMI. During US-guided axillary brachial plexus block local anesthetic should be injected around each of four nerves,^19^ whereas in the SCL approach injection of local solution should be performed directly into the sheath containing the nerves and also in the “corner pocket.”^17^ If the patient’s arm can be abducted to 90°, before the ICL approach for BPB, the triangular composition of all the three fascicles can be seen^3^,^25^ and the block can be performed quickly.

There was no difference between the groups with regard to a feeling of discomfort during the operation. Mild sedation can eliminate this discomfort.

In this study no serious complications were encountered (pneumothorax, temporary ipsilateral hemidiaphragmatic paresis with dyspnea and elevated hemidiaphragm, accidental vascular puncture, local anesthetic toxicity, prolonged neurologic deficit due to nerve damage). Transient Horner syndrome was diagnosed in three patients in the SCL group.

Although we used a high volume of the injected local anesthetic (40 mL) for each block in this study, it is possible that a smaller volume of local anesthetic can be used.

## CONCLUSIONS

- US-guided BPB can be performed by the three approaches, supra- or infraclavicular or axillary, with a similar quality of surgical anesthesia for operations of the upper extremity below the shoulder. An additional block of intercostobrachial and medial brachial cutaneous nerves is strongly recommended in all cases, regardless of the approach used.
- The time needed for performing a BPB by the axillary approach is slightly longer in comparison with the supraclavicular and infraclavicular approaches, but this small difference has no practical clinical significance.
- Obesity does not prolong the time of US-guided BPB performance.
- No serious complication of US-guided BPB was encountered in our study.

## Abbreviations

ASA: American Society of Anesthesiologists
AX: axillary approach for brachial plexus block, axillary block
BMI: body mass index
BPB: brachial plexus block
ICL: infraclavicular approach for brachial plexus block, infraclavicular block
INR: international normalized ratio
SCL: supraclavicular approach for brachial plexus block, supraclavicular block
US: ultrasound
